# Association between Fish Intake and Serum Testosterone Levels in Older Males: The Hitachi Health Study II

**DOI:** 10.1016/j.cdnut.2024.102133

**Published:** 2024-03-05

**Authors:** Aoi Ito, Shohei Yamamoto, Yosuke Inoue, Ami Fukunaga, Akiko Nanri, Maki Konishi, Shuichiro Yamamoto, Tohru Nakagawa, Tetsuya Mizoue

**Affiliations:** 1 Department of Epidemiology and Prevention, Center for Clinical Sciences, National Center for Global Health and Medicine, Tokyo, Japan; 2 Laboratory of Public Health, Graduate School of Integrated Pharmaceutical and Nutritional Sciences, University of Shizuoka, Shizuoka, Japan; 3 Department of Public Health and Health Policy, Graduate School of Biomedical and Health Sciences, Hiroshima University, Hiroshima, Japan; 4 Department of Food and Health Sciences, International College of Arts and Sciences, Fukuoka Women’s University, Fukuoka, Japan; 5 Hitachi Health Care Center, Hitachi, Ltd., Ibaraki, Japan

**Keywords:** testosterone, fatty fish, lean fish, older males, cross-sectional study

## Abstract

**Background:**

Fish are rich in omega-3 polyunsaturated fatty acids and vitamin D, which can promote testosterone synthesis and secretion. However, some contaminants present in fish may disrupt testosterone production.

**Objective:**

This study aimed to investigate the association between fish intake (total, fatty, and lean fish) and serum testosterone levels in older males.

**Methods:**

This cross-sectional study included 1545 Japanese males aged 60–69 y who participated in the baseline survey of the Hitachi Health Study II. Fish intake was estimated using a validated brief-type self-administered diet history questionnaire. Total testosterone levels were measured by chemiluminescence immunoassay. Multivariable linear regression analysis was used to analyze the association between fish intake and serum testosterone levels.

**Results:**

Higher total fish intake was associated with higher levels of serum testosterone, with an adjusted mean [95% confidence interval (CI) of 5.63 (5.43, 5.83) and 5.99 (5.78, 6.20)] ng/mL for the 1st and 4th quartiles of total fish intake, respectively (*P* for trend = 0.06). When analyzing fatty and lean fish separately, higher intake of lean fish, but not fatty fish, was associated with higher levels of serum testosterone: adjusted mean (95% CI): 5.63 (5.43, 5.82) and 6.00 (5.79, 6.20) ng/mL for the 1st and 4th quartiles of lean fish intake, respectively (*P* for trend = 0.01).

**Conclusions:**

Among older males, higher intake of total fish, particularly lean fish, was associated with higher serum testosterone levels. *Curr Dev Nutr* 20xx;x:xx.

## Introduction

Testosterone, a hormone secreted mainly by Leydig cells in the testis of males, plays an important role not only in regulating reproductive function but also in maintaining bone mineral density, muscle strength, and cognitive function [1]. Low testosterone levels are associated with higher risks of dementia [2], type 2 diabetes [3], and all-cause mortality in males [4]. Serum testosterone levels decline with age [5], but lifestyle modifications, such as increased physical activity can attenuate this decline in older males [6].

Diet may also affect serum concentrations of testosterone [7], which is synthesized from cholesterol through a series of enzymatic reactions and is regulated by pituitary gonadotropin luteinizing hormone [8]. Fish contains several key nutrients involved in testosterone synthesis and secretion, such as vitamin D [9], omega-3 polyunsaturated fatty acids (*n*-3 PUFAs) [10,11], and protein [12]. Animal experiments showed that *n*-3 PUFAs promoted luteinizing hormone-simulated testosterone synthesis by increasing the responsiveness of gonadotropin receptors in Leydig cells [10]. Vitamin D may enhance testosterone production in Leydig cells by regulating the expression of genes related to testosterone synthesis [9]. In humans, clinical trials have demonstrated that supplementation of fish oil rich in *n*-3 PUFAs [13,14] or vitamin D [15,16] increased serum testosterone levels in males. On the other hand, fish also contain environmental contaminants, including methylmercury and polychlorinated biphenyls (PCBs), which can inhibit serum testosterone production owing to their reproductive toxicity [[17], [18], [19], [20]].

Although fish contain both nutrients and contaminants that may affect serum testosterone levels differently, evidence is scarce for the association between fish intake and serum testosterone levels. Only one study of 178 middle-aged male boat captains and anglers in the United States showed that fish consumption was positively correlated with serum testosterone levels [19]. Furthermore, given that fatty fish typically contain higher amounts of *n*-3 PUFAs and vitamin D than lean fish [21], it is reasonable to assume that fatty fish intake is more strongly associated with serum testosterone levels than lean fish intake. No studies, however, examined the respective association of lean and fatty fish intake with serum testosterone levels.

This study aimed to examine the association between fish intake and serum testosterone levels among older males in Japan, one of the highest fish-consuming countries in the world and where a variety of fish is consumed [22,23].

## Methods

### Study design and participants

**FIGURE 1:**
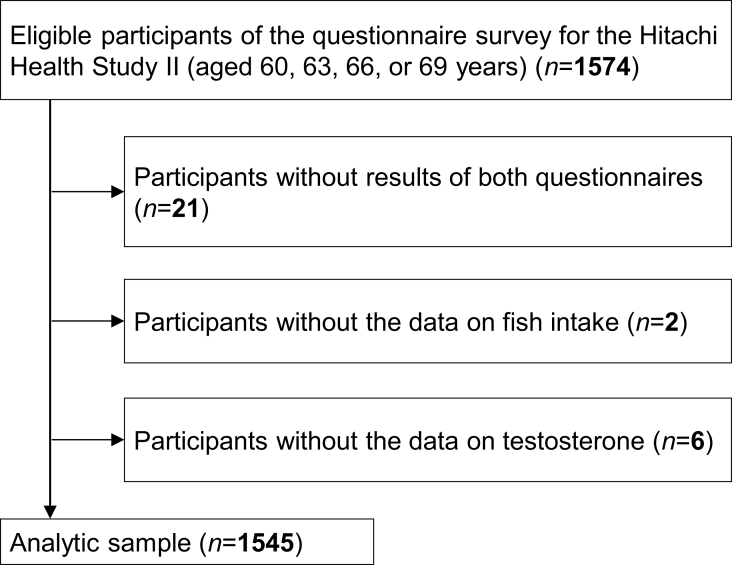
Flowchart of participants included in the present analysis.

Data for this cross-sectional study were obtained from the baseline survey of the Hitachi Health Study II, an ongoing prospective study of current and former employees and their spouses of Hitachi, Ltd., Japan [24,25]. A total of 3250 individuals (89.4% males) received a health check-up, including cognitive function screening, at the Hitachi Health Care Center (Ibaraki prefecture) between April 2017 and March 2020; of whom, 2101 were in their 60s. In each fiscal year, we invited participants aged 60, 63, 66, and 69 y (as of March 31, the last date of the fiscal year) to answer 2 questionnaires on overall health-related lifestyle and dietary habits, enabling us to cover those aged 60–69 y over the 3-y baseline period. Of 1663 health check-up examinees aged 60, 63, 66, and 69 y (95.7% males), 1574 males agreed and filled out the questionnaires. No female participated in the questionnaire survey. For this study, we excluded those without results from both questionnaires (*n* = 21) and those without the data on fish intake (*n* = 2) or testosterone levels (*n* = 6), leaving 1545 males for the analysis (Figure 1).

This study was conducted according to the guidelines laid down in the Declaration of Helsinki. All procedures involving human subjects were approved by the Ethics Committee of the National Center for Global Health and Medicine (approval number: NCGM-G-002208) and the Hitachi Health Care Center. Written informed consent was obtained from all participants before their participation in the present study.

### Dietary intake assessment

Information on dietary intake was assessed using the brief-type self-administered diet history questionnaire (BDHQ) [26,27]. The BDHQ assesses dietary habits during the preceding month and is designed to calculate the daily intake of commonly consumed 58 food items in Japan, including 38 solid foods (including 5 fish items), 12 beverages, and 8 seasonings. Fixed portion sizes of food items were derived from several recipe books for Japanese dishes. Fish intake was calculated based on the reported frequency of fish intake and the fixed portion size [26,27]. Total fish intake was calculated by summing the following 5 items: fatty fish (including sardines, mackerel, saury, amberjack, herring, eel, and fatty tuna), lean fish (including salmon, trout, white meat fish, freshwater fish, and bonito), dried fish and salted fish, small fish with bones, and canned tuna [27]. Shellfish was not included in the calculation of total fish intake. Salmon was classified as a lean fish because of the relatively low-fat content of salmon commonly consumed in Japan (for example, chum salmon, 3.7 g fat/100 g) [28]. Canned tuna was not incorporated in either the lean or fatty fish because it contains much lower amounts of fish-derived *n*-3PUFAs, including eicosapentaenoic acid and docosahexaenoic acid, than common fatty and lean fish [28,29]. Dietary intake of total energy, ethanol, and selected nutrients were assessed using an *ad hoc* computer algorithm for the BDHQ, according to the Standard Tables of Food Composition in Japan, 2010 [28]. Intakes of fish, *n*-3 PUFAs, vitamin D, cholesterol, magnesium, and zinc were energy-adjusted using the density method [30]. Protein (% energy) was calculated by multiplying protein intake (g/d) by 4 (Atwater factor), divided by total energy intake (kcal/d), and multiplied by 100.

The validity of the BDHQ for Japanese adults using semiweighted dietary records as the gold standard has been published elsewhere [27,31,32]; the Spearman’s correlation coefficients were 0.36 for energy-adjusted intake of fish and shellfish [27], and 0.27 for vitamin D [32]. The Pearson correlation coefficients were 0.38 for total energy intake, and that of energy-adjusted nutrient intake was 0.57 for protein, 0.41 for *n*-3 PUFA, 0.43 for cholesterol, 0.68 for magnesium, and 0.56 for zinc [31].

### Testosterone levels

Peripheral venous blood samples were collected between 8:00 and 10:00 a.m. and stored in freezers at −80°C until analysis. They were transferred to the laboratory (LSI Medience Co.) for measurement of serum total testosterone. Total testosterone was measured using the chemiluminescence immunoassay method with Architect testosterone II kits (Architect i2000 SR; Abbott). The intra-assay coefficient of variation of the kit is 2.0%–5.1% in samples with testosterone concentrations of 0.5–35 nmol/L (0.1–10.1 ng/mL). Low testosterone levels are defined as total testosterone concentrations <3.0 ng/mL based on the American Urological Association guideline [33].

### Other covariates

Information on marital status, education, employment status, household income, sleep duration during weekdays, and leisure-time physical activity was obtained using the study-specific questionnaire, whereas that on smoking status, comorbidities, and medication use was obtained using the questionnaire embedded in the health check-up. Leisure-time physical activity was expressed as the sum of metabolic equivalents (METs) multiplied by the duration of time engaged across activities with different intensity. Blood pressure was measured using an automatic sphygmomanometer. Body height and weight were measured to the nearest 0.1 kg and 0.1 cm, respectively. BMI was calculated as weight in kilograms divided by the squared height in meters. Hypertension was defined as systolic blood pressure ≥140 mmHg or diastolic blood pressure ≥90 mmHg based on the Japanese Society of Hypertension guidelines [34], or self-reported use of antihypertensive medication. Plasma glucose was measured using the glucose oxidase enzyme-electrode method (A&T), and hemoglobin A1c (HbA1c) was measured using the high-performance liquid chromatography method (HLC723-G9, TOSOH). Diabetes was defined as fasting plasma glucose ≥126 mg/dL or HbA1c ≥6.5% based on the American Diabetes Association criteria [35]. Those receiving anti-diabetic treatment were also classified as having diabetes.

### Treatment of missing data

The proportion of missing data for each covariate was as follows: marital status: 1.4%, education: 0.1%, employment status: 3.5%, diabetes: 0.3%, use of cholesterol/triglyceride-lowering medication: 26.3%, household income: 1.0%, sleep duration: 0.4%, and physical activity: 5.9%. Multiple imputations for missing data were performed using the chained equation method, assuming that analyzed data were missing at random [36]. All variables used in the analysis were included in the creation of 20 imputed datasets with predictive mean matching (the closest 3 observations were drawn). Each imputed dataset was analyzed, and the results across imputations were combined using Rubin’s rules [37].

### Statistical analysis

We compared the characteristics of study participants across the quartiles of energy-adjusted intake of total, fatty, and lean fish. Multivariable linear regression analysis was performed to estimate and compare the adjusted means of total testosterone levels at each quartile of intake of total, lean, and fatty fish. Model 1 was adjusted for age (y, continuous). Model 2 was additionally adjusted for marital status (married or others), education (<10, 10–12, or ≥13 y), current work (employed or unemployed), household income (<3, 3–5.9, or ≥6 million Japanese yen/y), BMI (kg/m^2^, continuous), smoking status (never, former, or current), use of cholesterol- or triglyceride-lowering drugs (yes or no), alcohol consumption (nondrinkers; drinkers consuming <23, 23–45.9, or ≥46 g ethanol/d), sleep duration (<7, 7–7.9, or ≥8 h/d), leisure-time physical activity (METs h/wk, quartile), total energy intake (kcal/d, continuous), cholesterol intake (mg/1000 kcal, continuous), zinc intake (mg/1000 kcal, continuous), and magnesium intake (mg/1000 kcal, continuous). These covariates were selected based on previous literature [6,8,[38], [39], [40]]. In the analysis examining the association between lean or fatty fish intake and serum testosterone levels, the model was further adjusted for the combined intake of salted fish and dried fish, small fish with bones, and canned tuna (which were classified as neither lean nor fatty fish), as well as for lean or fatty fish for mutual adjustment. The *P* value for the trend was calculated by treating the quartile variable of fish intake as a continuous term in the model. Sensitivity analysis was performed to investigate the potential impact of very high fish intake on the association by excluding participants with a total fish intake exceeding 100 g/1000 kcal (*n* = 20).

To investigate whether a dose-response association existed between fish intake and predicted serum testosterone levels, we ran the model while treating fish intake as a cubic polynomial (that is, in terms of fish intake, fish intake^2^, and fish intake^3^). All data on fish intake were used in the analysis, but data were truncated at 108.5 g/1000 kcal for total fish, 46.3 g/1000 kcal for lean fish, and 35.2 g/1000 kcal for fatty fish (99% of the distribution) because of the limited number of data when making a cubic spline plot. *P* value for linearity was calculated by treating fish intake as a continuous variable in the model. Logistic regression was also performed to calculate the odds ratios (OR) and 95% confidence interval (95% CI) for having low testosterone levels (<3.0 ng/mL) across the quartiles of fish intake. All statistical analyses were conducted using Stata ver. 18.0 (StataCorp LLC, College Station). All the reported *P* values were 2-tailed, and statistical significance was set at *P* <0.05.

## Results

**FIGURE 2:**
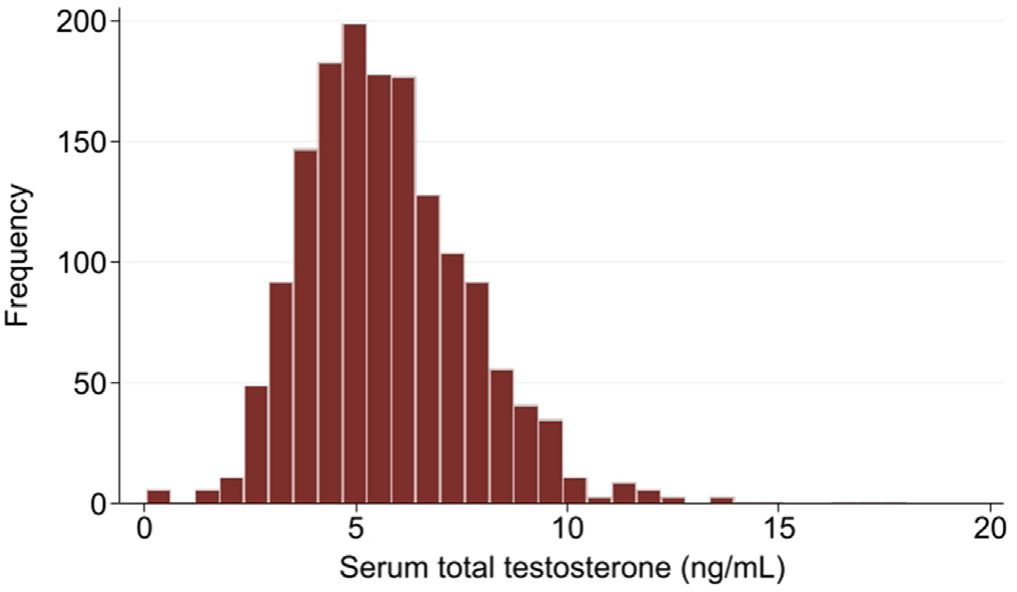
Distribution of serum total testosterone levels of 1545 males aged 60–69 y.x

The participants had a mean (standard deviation [SD]) age of 63.5 (3.5) y and a mean (SD) serum total testosterone level of 5.7 (2.0) ng/mL. The distribution of serum total testosterone levels is shown in Figure 2. The median (interquartile range [IQR]) intake of total, fatty, lean, and other fish (including dried fish and salted fish, small fish with bones, and canned tuna) was 29 (20–45), 7 (4–11), 7 (4–12), and 13 (8–21) g/1000 kcal, respectively. Participants with a higher intake of total fish tended to sleep longer and have a higher intake of total energy, cholesterol, protein, vitamin D, *n*-3 PUFAs, zinc, and magnesium (Table 1). Similar results were obtained when comparing the characteristics across the quartiles of lean and fatty fish (Supplemental Tables 1 and 2).

**TABLE 1:** Participant characteristics

	Total	Total fish intake			
		Q1	Q2	Q3	Q4
Participants, *n*	1545	387	386	386	386
Total fish (g/1000 kcal) [median (min–max)]		14 (0–19.7)	24 (19.8–29.4)	36 (29.5–44.6)	58 (44.9–162.9)
Age (y)	63.5 (3.5)	63.5 (3.5)	63.3 (3.5)	63.4 (3.4)	63.9 (3.5)
Marital status, married	90.6	90.8	91.1	88.7	91.6
Education, <10 y	5.0	4.9	4.4	2.6	8.0
Employment status, employed	71.4	73.0	71.1	70.8	70.7
Household income, <3 million Japanese yen/y	30.3	29.1	27.2	33.9	30.9
Current use of cholesterol/triglyceride-lowering medication	23.4	25.8	20.9	23.1	24.0
Comorbidities					
Diabetes^1^	23.2	24.4	22.0	21.4	25.1
Hypertension^2^	48.2	47.0	47.7	48.2	50.0
Dyslipidemia^3^	59.0	63.8	58.8	59.1	54.4
Cancer	6.6	5.7	8.0	7.8	4.9
Chronic kidney disease	1.3	1.6	1.6	0.8	1.3
Cardiovascular disease	11.8	12.4	10.6	12.2	12.2
BMI (kg/m^2^)	24.2 (3.1)	24.4 (3.0)	23.9 (2.9)	24.4 (3.5)	24.1 (3.1)
Smoking status, current	22.1	22.7	23.6	23.1	19.2
Sleep duration, <7 h/d	37.4	40.3	39.5	36.1	33.6
Physical activity on leisure, ≥10 METs h/wk	35.0	34.2	34.8	35.6	35.5
Dietary intake (/d)					
Total energy (kcal)	1957 (554)	1821 (554)	1942 (506)	2005 (552)	2060 (574)
Alcohol (g) [median (IQR)]	12.1 (1.0–30.1)	10.2 (0.2–30.1)	12.0 (1.0–28.7)	14.3 (1.2–33.4)	11.9 (1.2–28.6)
Protein (% energy)	14.8 (2.9)	12.7 (2.3)	13.8 (2.0)	15.0 (1.9)	17.8 (2.6)
Cholesterol (mg/1000 kcal)	195.3 (74.0)	167.3 (79.7)	180.8 (62.7)	192.3 (60.0)	240.8 (70.6)
*n*-3 PUFAs (g/1000 kcal)	1.4 (0.5)	1.0 (0.3)	1.2 (0.3)	1.5 (0.3)	1.9 (0.4)
Vitamin D (μg/1000 kcal)	7.2 (4.4)	3.2 (1.1)	5.3 (0.9)	7.6 (1.3)	13.0 (4.3)
Zinc (mg/1000 kcal)	4.3 (0.7)	4.1 (0.7)	4.2 (0.6)	4.3 (0.6)	4.7 (0.6)
Magnesium (mg/1000 kcal)	136.7 (28.2)	124.3 (26.4)	129.6 (25.8)	138.1 (22.4)	154.7 (28.1)

Data are presented as percentages or mean (standard deviation) except where noted.

Abbreviations: BMI, body mass index; IQR, inter quartile range; HbA1c, hemoglobin A1c; MET, metabolic equivalent; Q, quartile.

1 Defined as fasting plasma glucose ≥126 mg/dL, HbA1c ≥6.5%, and/or self-reported medical history or medication use for diabetes.

2 Defined as systolic blood pressure ≥140 mmHg, diastolic blood pressure ≥90 mmHg, and/or self-reported medical history or medication use for hypertension.

3 Defined as low-density lipoprotein-cholesterol ≥140 mg/dL, triglyceride ≥150 mg/dL, high-density lipoprotein-cholesterol <40 mg/dL, and/or self-reported medical history or medication use for dyslipidemia.

**FIGURE 3:**
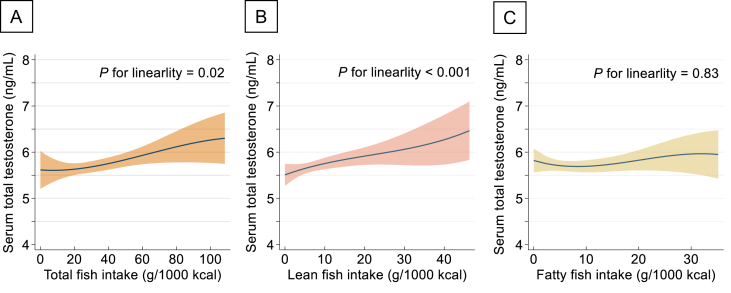
Cubic spline curve plot of the association between fish intake and serum testosterone levels. (A) total fish, (B) lean fish, and (C) fatty fish. Solid line represents the predicted levels of serum testosterone based on observed data of fish intake, and colored background represents its 95% confidence intervals. The model (Model 2) is adjusted for age (y, continuous), marital status (married or others), education (<10, 10–12, or ≥13 y), employment status (employed or unemployed), household income (<3, 3–5,9, or ≥6 million Japanese yen/y), BMI (kg/m^2^, continuous), smoking status (never, former, or current), use of cholesterol- or triglyceride-lowering drugs (yes or no), alcohol consumption (nondrinkers; drinkers consuming <23, 23–45.9, or ≥46 g ethanol/d), sleep duration (<7, 7–7.9, or ≥8 h/d), leisure-time physical activity (metabolic equivalents h/wk, quartile), energy intake (kcal/d, continuous), cholesterol intake (mg/1000 kcal, continuous), zinc intake (mg/1000 kcal, continuous), and magnesium intake (mg/1000 kcal, continuous). In the separate analysis of lean and fatty fish, the model was further adjusted for the combined intake of salted fish and dried fish, small fish with bones, and canned tuna, as well as for lean or fatty fish for mutual adjustment.

Higher intake of total fish was associated with higher levels of serum testosterone; the adjusted means (95% CIs) were 5.63 (5.43, 5.83) and 5.99 (5.78, 6.20) ng/mL for the 1st and 4th quartiles of total fish intake, respectively (Model 2: *P* for trend = 0.06, Table 2). These associations were virtually unchanged after excluding those with a total fish intake of ≥100 g/1000 kcal (Supplemental Table 3). When analyzing fatty and lean fish separately, a higher intake of lean fish, but not fatty fish, was associated with higher levels of serum testosterone; the adjusted means (95% CIs) were 5.63 (5.43, 5.82) and 6.00 (5.79, 6.20) ng/mL for the 1st and 4th quartiles of lean fish intake, respectively (Model 2: *P* for trend = 0.01, Table 2). Spline analysis showed a steady increase in serum testosterone levels with increasing total fish intake or lean fish intake (*P* for linearity for total fish = 0.02, Figure 3A; *P* for linearity for lean fish <0.001, Figure 3B). Higher total or lean fish intake was associated with lower, albeit not statistically significant, odds of low testosterone levels (<3.0 ng/mL): ORs (95% CI) for the highest intake of total fish and lean fish were 0.54 (0.25, 1.16) and 0.70 (0.33, 1.47), respectively (Supplemental Table 4).

**TABLE 2:** Adjusted means of serum testosterone levels and their 95% confidence intervals by quartiles of energy-adjusted fish intake

	Q1 (*n* = 387)	Q2 (*n* = 386)	Q3 (*n* = 386)	Q4 (*n* = 386)	*P* for trend^1^
Total fish, median (min–max) (g/1000 kcal)	14 (0, 19.7)	24 (19.8, 29.4)	36 (29.5, 44.6)	58 (44.9, 162.9)	
Serum total testosterone levels (ng/mL), adjusted mean (95% CI)^2^					
Model 1^3^	5.58 (5.38, 5.79)	5.79 (5.59, 5.99)	5.57 (5.37, 5.77)	5.99 (5.79, 6.19)^5^	0.03
Model 2^4^	5.63 (5.43, 5.83)	5.71 (5.51, 5.90)	5.62 (5.42, 5.81)	5.99 (5.78, 6.20)^5^	0.06
Lean fish, median (min–max) (g/1000 kcal)	3 (0, 4.26)	6 (4.28, 7.29)	9 (7.31, 12.1)	19 (12.2, 75.5)	
Serum total testosterone levels (ng/mL), adjusted mean (95% CI)^2^					
Model 1^3^	5.58 (5.38, 5.79)	5.60 (5.39, 5.80)	5.76 (5.56, 5.96)	6.00 (5.80, 6.20)^5^	<0.01
Model 2^4^^,^^6^	5.63 (5.43, 5.82)	5.60 (5.41, 5.79)	5.71 (5.52, 5.91)	6.00 (5.79, 6.20)^5^	0.01
Fatty fish, median (min–max) (g/1000 kcal)	3 (0, 4.2)	6 (4.3, 7.2)	9 (7.3, 10.9)	17 (11.0, 57.4)	
Serum total testosterone levels (ng/mL), adjusted mean (95% CI)^2^					
Model 1^3^	5.72 (5.52, 5.92)	5.70 (5.50, 5.90)	5.69 (5.49, 5.90)	5.82 (5.62, 6.02)	0.52
Model 2^4^^,^^6^	5.78 (5.59, 5.98)	5.73 (5.54, 5.92)	5.65 (5.46, 5.85)	5.77 (5.57, 5.97)	0.77

The number of participants in each quartile is the same for total, lean, and fatty fish intake.

Abbreviation: CI, confidence interval; MET, metabolic equivalent; Q, quartile.

1 *P* value for the trend is calculated by treating the quartiles of fish intake as a continuous term in linear regression model.

2 Estimated adjusted means and its 95% confidence interval of serum total testosterone level (ng/mL).

3 Adjusted for age (y, continuous).

4 Adjusted for age (y, continuous), marital status (married or others), education (<10, 10–12, or ≥13 y), employment status (employed or unemployed), household income (<3, 3–5, 9, or ≥6 million Japanese yen/y), BMI (kg/m^2^, continuous), smoking status (never, former, or current), use of cholesterol- or triglyceride-lowering drugs (yes or no), alcohol consumption (nondrinkers; drinkers consuming <23, 23–45.9, or ≥46 g ethanol/d), sleep duration (<7, 7–7.9, or ≥8 h/d), leisure-time physical activity (METs h/wk, quartile), energy intake (kcal/d, continuous), cholesterol intake (mg/1000 kcal, continuous), zinc intake (mg/1000 kcal, continuous), and magnesium intake (mg/1000 kcal, continuous).

5 The values show the quartile of fish intake significantly associated with serum testosterone levels, with the 1st quartile as a reference (*P* value <0.05).

6 Further adjusted for the combined intake of salted fish and dried fish, small fish with bones, and canned tuna, as well as for lean or fatty fish for mutual adjustment.

## Discussion

Among older Japanese males, those with a higher intake of total fish had higher serum testosterone levels. When analyzing the association in relation to fatty and lean fish intake separately, we found that a higher intake of lean fish, but not fatty fish, was associated with higher levels of serum testosterone.

A previous study of 178 middle-aged United States males showed a positive correlation between sport-caught fish consumption and serum testosterone levels, with no adjustment for other dietary and nondietary lifestyle factors [19]. Compared with the participants of the United States study, those in the present study are older (weighted mean: 49.4 y compared with mean 63.5 y) and leaner (weighted mean: 27.2 kg/m^2^ compared with mean: 24.2 kg/m^2^) and had higher testosterone concentrations (weighted mean: 3.9 ng/mL compared with mean: 5.7 ng/mL). The present study of Japanese males in their 60s expands the previous United States research regarding sport-caught fish consumption by linking circulating testosterone to the usual consumption of fish while accounting for known lifestyle determinants, including physical activity and sleep duration.

Fish is a major dietary source of *n*-3 PUFAs, vitamin D, and protein, which potentially enhances testosterone synthesis [[9], [10], [11], [12]]. These nutrients might underlie the association between fish intake and serum testosterone levels. In male rats, a diet high in *n*-3 PUFAs enhanced luteinizing hormone-stimulated testosterone synthesis [10]. In humans, *n*-3 PUFAs-enriched fish oil supplementation resulted in elevated total testosterone levels in males [13], and another study also found a similar, albeit not statistically significant, increase in total testosterone levels [14]. Vitamin D enhances testosterone synthesis in Leydig cells of male mice [9]. A few human studies [15,16,41], but not all [42,43], showed vitamin D supplementation increased serum total testosterone levels. Regarding protein, a meta-analysis of animal studies found that a low-protein diet reduced testosterone levels [12], possibly because of hypothalamic-pituitary-gonadal axis dysfunction [44].

Contrary to our expectations, we found no association between fatty fish intake and serum testosterone levels. One possible explanation for this finding is that environmental contaminants present in fatty fish offset the promoting effects of *n*-3 PUFAs and vitamin D on testosterone synthesis. Animal experiments showed that some contaminants, such as dioxin and PCBs, which are more concentrated in fatty fish than in lean fish [45,46], reduced serum testosterone levels [47,48]. Among Japanese adults, a higher intake of fatty coastal fish (for example, mackerel and sardine) is associated with higher blood levels of dioxins and PCBs [49]. Some population-based studies in the United States also showed that serum PCB levels were inversely associated with serum testosterone levels [[50], [51], [52]].

In males, higher serum testosterone levels have been associated with lower fat mass, higher bone mineral density, and lower insulin resistance [6]. A clinical trial in males demonstrated that fish oil supplementation increased serum testosterone levels by 0.56 ng/mL, and this change was associated with improved insulin resistance [13]. Another clinical trial among overweight males showed that vitamin D supplements increased serum testosterone levels by 0.78 ng/mL and decreased BMI by 2.1 kg/m^2^ [15]. The difference in serum testosterone levels between the lowest and highest quartiles of total fish intake (0.36 ng/mL) in our study is somewhat smaller than the changes reported from these supplementation studies; it is thus unclear whether such a modest difference has clinical significance. In our explanatory analysis, however, there was a suggestion of decreased odds of clinically defined low testosterone (<3.0 ng/mL) associated with the highest fish intake.

This study has some limitations. First, the association derived from a cross-sectional study does not necessarily indicate causality, although it is unlikely that serum testosterone levels influence fish intake. Second, the possibility of bias cannot be ruled out because of residual confounding and unmeasured factors (for example, contaminants derived from fish and fish size). Consumption of larger fish, which are higher up the food chain and contain higher concentrations of environmental contaminants, may influence serum testosterone levels. Additional adjustments for these factors may provide a better understanding of the association between fish intake and serum testosterone levels. Third, fish intake was self-reported; thus, there was a possibility of misreporting in the type of fish consumed and its frequency. Fourth, we only had data on total testosterone levels. Free testosterone level, the bioavailable form of testosterone, may more accurately reflect the circulating testosterone levels of older Japanese males [53]. Finally, the study participants were current or retired employees of 1 manufacturer in Japan, and thus, the present findings might not be generalizable to populations with different background characteristics.

In conclusion, this cross-sectional study showed that a higher intake of total or lean fish was associated with higher serum testosterone levels among older Japanese males. Future studies with measurements of specific biomarkers of fish consumption (for example, *n*-3 PUFAs) and environmental contaminants can contribute to a better understanding of the association between fish intake and serum testosterone levels.

## Author contributions

The authors’ responsibilities were as follows – All authors: contributed to the conception, design, and interpretation of the data; AI contributed to the data analysis; TN, ShuY: contributed to the acquisition of data; AI: drafted the manuscript; ShoY, AN, AF, YI, TN, ShuY, TM: contributed to the critical revision of the manuscript; AI: had full access to all the data in the study and took responsibility for the integrity of the data and the accuracy of the data analysis; and all authors: read and agreed to the published version of the manuscript.

### Conflict of interest

The authors report no conflicts of interest.

### Funding

This work was supported by the National Center for Global Health and Medicine (19A1006, 21A1020, 22A1008). The funding sources had no role in the design, analysis, or preparation of this manuscript.

### Data availability

The datasets generated and/or analyzed during the current study are not publicly available because of ethical restrictions and participant confidentiality concerns, but de-identified data are available from TM to qualified researchers on reasonable request.

## Appendix A Supplementary data

Multimedia component1 Multimedia component1

## References

[1] Kang H.-Y. (2013). Beyond the male sex hormone: deciphering the metabolic and vascular actions of testosterone. J. Endocrinol..

[2] Zhang Z., Kang D., Li H. (2021). Testosterone and cognitive impairment or dementia in middle-aged or aging males: causation and intervention, a systematic review and meta-analysis. J. Geriatr. Psychiatry Neurol..

[3] Yao Q.-M., Wang B., An X.-F., Zhang J.-A., Ding L. (2018). Testosterone level and risk of type 2 diabetes in men: a systematic review and meta-analysis. Endocr. Connect..

[4] Araujo A.B., Dixon J.M., Suarez E.A., Murad M.H., Guey L.T., Wittert G.A. (2011). Endogenous testosterone and mortality in men: a systematic review and meta-analysis. J. Clin. Endocrinol. Metab..

[5] Harman S.M., Metter E.J., Tobin J.D., Pearson J., Blackman M.R. (2001). Longitudinal effects of aging on serum total and free testosterone levels in healthy men. J. Clin. Endocrinol. Metab..

[6] Kaufman J.-M., Lapauw B., Mahmoud A., T’Sjoen G., Huhtaniemi I.T. (2019). Aging and the male reproductive system. Endocr. Rev..

[7] Allen N.E., Key T.J. (2000). The effects of diet on circulating sex hormone levels in men. Nutr. Res. Rev..

[8] Payne A.H., Hales D.B. (2004). Overview of steroidogenic enzymes in the pathway from cholesterol to active steroid hormones. Endocr. Rev..

[9] Wang L., Lu H., Wang S., Liu H., Guo M., Bai H. (2022). Vitamin D Receptor affects male mouse fertility via regulation of lipid metabolism and testosterone biosynthesis in testis. Gene.

[10] Sebokova E., Garg M.L., Wierzbicki A., Thomson A.B.R., Clandinin M.T. (1990). Alteration of the lipid composition of rat testicular plasma membranes by dietary (n-3) fatty acids changes the responsiveness of Leydig cells and testosterone synthesis. J. Nutr..

[11] Zaima N., Kinoshita S., Hieda N., Kugo H., Narisawa K., Yamamoto A. (2016). Effect of dietary fish oil on mouse testosterone level and the distribution of eicosapentaenoic acid-containing phosphatidylcholine in testicular interstitium. Biochem. Biophys. Rep..

[12] Ajuogu P.K., Al-Aqbi M.A., Hart R.A., Wolden M., Smart N.A., McFarlane J.R. (2020). The effect of dietary protein intake on factors associated with male infertility: a systematic literature review and meta-analysis of animal clinical trials in rats. Nutr. Health..

[13] Abbott K., Burrows T.L., Acharya S., Thota R.N., Garg M.L. (2020). Dietary supplementation with docosahexaenoic acid rich fish oil increases circulating levels of testosterone in overweight and obese men. Prostaglandins Leukot. Essent. Fatty Acids..

[14] Hughes G.S., Ringer T.V., Watts K.C., DeLoof M.J., Francom S.F., Spillers C.R. (1990). Fish oil produces an atherogenic lipid profile in hypertensive men. Atherosclerosis.

[15] Pilz S., Frisch S., Koertke H., Kuhn J., Dreier J., Obermayer-Pietsch B. (2011). Effect of vitamin d supplementation on testosterone levels in men. Horm. Metab. Res..

[16] Canguven O., Talib R.A., El Ansari W., Yassin D.-J., Al Naimi A. (2017). Vitamin D treatment improves levels of sexual hormones, metabolic parameters and erectile function in middle-aged vitamin D deficient men. Aging Male.

[17] Serag El Din O.S., Abd El Azim B.H., Lotfy R.A. (2018). Fish diet and male reproductive hormones in albino rats. J. Basic Appl. Zool..

[18] Gdoura N., Abdelmouleh A., Chaabouni K., Ayadi F.M., Guermazi F., Murat J.-C. (2011). Alteration of male reproductive system in rats fed on red or white meat from tuna ﬁsh caught in the Gulf of Gabe’s in Tunisian coast. Environ. Chem. Lett..

[19] Persky V., Turyk M., Anderson H.A., Hanrahan L.P., Falk C., Steenport D.N. (2001). The effects of PCB exposure and fish consumption on endogenous hormones. Environ. Health Perspect..

[20] Dhooge W., Van Larebeke N., Koppen G., Nelen V., Schoeters G., Vlietinck R. (2006). Serum dioxin-like activity is associated with reproductive parameters in young men from the general flemish population. Environ. Health Perspect..

[21] Weichselbaum E., Coe S., Buttriss J., Stanner S. (2013). Fish in the diet: a review. Nutr. Bull..

[22] Kamoey A. (2015). The Japanese market for seafood. Globefish Res. Program..

[23] FAO (2020). Sustainability in Action.

[24] Fujiwara A., Fukunaga A., Murakami K., Inoue Y., Nakagawa T., Yamamoto S. (2023). Cross-sectional association between estimated hardness of the habitual diet and depressive symptoms in older Japanese men. Nutrients.

[25] Fujiwara A., Fukunaga A., Murakami K., Inoue Y., Nakagawa T., Yamamoto S. (2023). Association between dietary hardness and cognitive dysfunction among Japanese men in their 60s: a cross-sectional study. Nutrients.

[26] Sasaki S., Yanagibori R., Amano K. (1998). Self-administered diet history questionnaire developed for health education: a relative validation of the test-version by comparison with 3-day diet record in women. J. Epidemiol..

[27] Kobayashi S., Murakami K., Sasaki S., Okubo H., Hirota N., Notsu A. (2011). Comparison of relative validity of food group intakes estimated by comprehensive and brief-type self-administered diet history questionnaires against 16 d dietary records in Japanese adults. Public Health Nutr.

[28] Ministry of Education (2010).

[29] Harauma A., Wakinaka N., Takeda M., Moriguchi T. (2017). Commercial tuna can is not a source of omega-3 fatty acids. J. Lipid. Nutr..

[30] W. Willett, Nutritional Epidemiology, 3rd ed, Oxford University Press, United Kingdom (2013), 10.1093/acprof:oso/9780199754038.001.0001.

[31] Kobayashi S., Honda S., Murakami K., Sasaki S., Okubo H., Hirota N. (2012). Both comprehensive and brief self-administered diet history questionnaires satisfactorily rank nutrient intakes in Japanese adults. J. Epidemiol..

[32] Kobayashi S., Yuan X., Sasaki S., Osawa Y., Hirata T., Abe Y. (2019). Relative validity of brief-type self-administered diet history questionnaire among very old Japanese aged 80 years or older. Public Health Nutr.

[33] Mulhall J.P., Trost L.W., Brannigan R.E., Kurtz E.G., Redmon J.B., Chiles K.A. (2018). Evaluation and management of testosterone deficiency: AUA guideline. J. Urol..

[34] Umemura S., Arima H., Arima S., Asayama K., Dohi Y., Hirooka Y. (2019). The Japanese Society of Hypertension Guidelines for the Management of Hypertension (JSH 2019). Hypertens. Res..

[35] American Diabetes Association, 2 (2021). Classification and diagnosis of diabetes: standards of medical care in diabetes – 2021. Diabetes Care.

[36] Van Buuren S., Boshuizen H.C., Knook D.L. (1999). Multiple imputation of missing blood pressure covariates in survival analysis. Stat. Med..

[37] Rubin D.B. (1987). Multiple Imputation for Nonresponse in Surveys.

[38] Te L., Liu J, Ma J, Wang S (2023). Correlation between serum zinc and testosterone: a systematic review. J. Trace Elem. Med. Biol..

[39] Roney J.R., Gettler L.T. (2015). The role of testosterone in human romantic relationships. Curr. Opin. Psychol..

[40] Wrzosek M., Woźniak J., Włodarek D. (2020). The causes of adverse changes of testosterone levels in men. Expert Rev. Endocrinol. Metab..

[41] Scholten S.D., Sergeev I.N., Song Q., Birger C.B. (2015). Effects of vitamin D and quercetin, alone and in combination, on cardiorespiratory fitness and muscle function in physically active male adults. Open Access J. Sports Med..

[42] Heijboer A.C., Oosterwerff M., Schroten N.F., Eekhoff E.M.W., Chel V.G.M., De Boer R.A. (2015). Vitamin D supplementation and testosterone concentrations in male human subjects. Clin. Endocrinol. (Oxf)..

[43] Lerchbaum E., Trummer C., Theiler-Schwetz V., Kollmann M., Wölfler M., Heijboer A.C. (2019). Effects of vitamin D supplementation on androgens in men with low testosterone levels: a randomized controlled trial. Eur. J. Nutr..

[44] De Oliveira J.C., De Moura E.G., Miranda R.A., De Moraes A.M.P., Barella L.F., Da Conceição E.P.S. (2018). Low-protein diet in puberty impairs testosterone output and energy metabolism in male rats. J. Endocrinol..

[45] Nøstbakken O.J., Rasinger J.D., Hannisdal R., Sanden M., Frøyland L., Duinker A. (2021). Levels of omega 3 fatty acids, vitamin D, dioxins and dioxin-like PCBs in oily fish; a new perspective on the reporting of nutrient and contaminant data for risk-benefit assessments of oily seafood. Environ. Int..

[46] Thutsumi T., Amakura Y., Fukuzawa E., Kono Y., Nakamura M., Nomura T. (2010). Dioxins in fish and shellfish: concentrations and intake in Japan. Organohalogen. Compd..

[47] Andric S.A., Kostic T.S., Stojilkovic S.S., Kovacevic R.Z. (2000). Inhibition of rat testicular androgenesis by a polychlorinated biphenyl mixture Aroclor 12481. Biol. Reprod..

[48] Zhang T., Zhou X., Ren X., Zhang X., Wu J., Wang S. (2021). Animal toxicology studies on the male reproductive effects of 2,3,7,8-tetrachlorodibenzo-p-dioxin: data analysis and health effects evaluation. Front. Endocrinol..

[49] Arisawa K., Matsumura T., Tohyama C., Saito H., Satoh H., Nagai M. (2003). Fish intake, plasma omega-3 polyunsaturated fatty acids, and polychlorinated dibenzo-p-dioxins/polychlorinated dibenzo-furans and co-planar polychlorinated biphenyls in the blood of the Japanese population. Int. Arch. Occup. Environ. Health..

[50] Leong J.Y., Blachman-Braun R., Patel A.S., Patel P., Ramasamy R. (2019). Association between polychlorinated biphenyl 153 exposure and serum testosterone levels: analysis of the National Health and Nutrition Examination Survey. Transl. Androl. Urol..

[51] Goncharov A., Rej R., Negoita S., Schymura M., Santiago-Rivera A., Morse G. (2009). Lower serum testosterone associated with elevated polychlorinated biphenyl concentrations in Native American men. Environ. Health Perspect..

[52] Ferguson K.K., Hauser R., Altshul L., Meeker J.D. (2012). Serum concentrations of p, p’-DDE, HCB, PCBs and reproductive hormones among men of reproductive age. Reprod. Toxicol..

[53] Okamura K., Ando F., Shimokata H. (2005). Serum total and free testosterone level of Japanese men: a population-based study. Int. J. Urol..

