# Supplementary material for: Association between Fish Intake and Serum Testosterone Levels in Older Males: The Hitachi Health Study II
Source: Curr Dev Nutr. 2024 Mar 5;8(4):102133. doi: 10.1016/j.cdnut.2024.102133 (PMC10997911; doi:10.1016/j.cdnut.2024.102133)
Supplement: Multimedia component1 [file mmc1.docx]

**Association between fish intake and serum testosterone levels in older males: The Hitachi Health Study II**

**Aoi Ito**

**Supplementary Table 1.** Participant characteristics according to the quartiles of lean fish

|  | **Lean fish intake** | | | |
| --- | --- | --- | --- | --- |
|  | **Q1** | **Q2** | **Q3** | **Q4** |
| **Participants**, *n* | 387 | 386 | 386 | 386 |
| **Lean fish**, g/1000 kcal (median [min–max]) | 3 (0–4.26) | 6 (4.28–7.29) | 9 (7.31–12.1) | 19 (12.2–75.5) |
| **Age**, years | 63.4 (3.5) | 63.7 (3.5) | 63.1 (3.4) | 63.9 (3.4) |
| **Marital status**, married | 88.1 | 91.6 | 91.6 | 90.8 |
| **Education**, <10 years | 4.4 | 3.9 | 4.9 | 6.7 |
| **Employment status**, employed | 71.5 | 69.7 | 74.5 | 70.1 |
| **Household income**, <3 million Japanese yen/year | 30.8 | 31.7 | 27.4 | 31.2 |
| **Current use of cholesterol/triglyceride-lowering medication** | 24.3 | 24.4 | 20.8 | 24.2 |
| **Comorbidities** |  |  |  |  |
| **Diabetes**^1^ | 22.5 | 22.4 | 23.9 | 24.1 |
| **Hypertension**^2^ | 48.1 | 51.0 | 43.3 | 50.5 |
| **Dyslipidemia**^3^ | 63.3 | 60.4 | 59.1 | 53.4 |
| **Cancer** | 7.0 | 5.7 | 7.3 | 6.5 |
| **Chronic kidney disease** | 1.6 | 1.0 | 0.8 | 1.8 |
| **Cardiovascular disease** | 11.9 | 11.4 | 9.6 | 14.5 |
| **BMI**, kg/m^2^ | 24.4 (3.0) | 24.2 (3.0) | 24.1 (3.2) | 24.2 (3.3) |
| **Smoking status**, current | 25.1 | 21.5 | 23.6 | 18.4 |
| **Sleep duration**, <7 hours/day | 36.0 | 40.1 | 37.5 | 35.8 |
| **Physical activity on leisure**, ≥10 METs･h/week | 34.3 | 35.5 | 31.6 | 38.7 |
| **Dietary intake**, per day |  |  |  |  |
| **Total energy**, kcal | 1958 (559) | 1972 (598) | 1790 (428) | 2108 (570) |
| **Protein**, % energy | 13.5 (2.7) | 14.0 (2.5) | 14.9 (2.5) | 16.9 (2.7) |
| **Cholesterol**, mg/1000 kcal | 179.3 (81.1) | 185.8 (69.8) | 194.5 (68.4) | 221.5 (69.1) |
| **Alcohol**, g (median [IQR]) | 12.1 (0.2-31.7) | 12.8 (1.2-33.1) | 9.6 (0.2-27.0) | 14.5 (2.4-33.2) |
| ***n*-3 PUFAs**, g/1000 kcal | 1.2 (0.4) | 1.3 (0.4) | 1.4 (0.4) | 1.7 (0.4) |
| **Vitamin D**, μg/1000 kcal | 4.9 (3.5) | 5.9 (3.1) | 7.3 (3.9) | 10.8 (4.3) |
| **Zinc**, mg/1000 kcal | 4.2 (0.7) | 4.2 (0.6) | 4.4 (0.6) | 4.5 (0.6) |
| **Magnesium**, mg/1000 kcal | 128.7 (28.2) | 133.0 (26.2) | 137.7 (28.0) | 147.3 (26.8) |

Data are presented as percentages or mean (standard deviation) except where noted.

^1^Defined as fasting plasma glucose ≥126 mg/dL, HbA1c ≥6.5%, and/or self-reported medical history or medication use for diabetes.
^2^Defined as systolic blood pressure ≥140 mmHg, diastolic blood pressure ≥ 90 mmHg, and/or self-reported medical history or medication use for hypertension.
^3^Defined as low-density lipoprotein-cholesterol ≥140 mg/dL, triglyceride ≥150 mg/dL, high-density lipoprotein-cholesterol <40 mg/dL, and/or self-reported medical history or medication use for dyslipidemia.
BMI, body mass index; IQR, inter quartile range; MET, metabolic equivalent; *n*-3 PUFAs, omega-3 polyunsaturated fatty acids; Q, quartile; SD, standard deviation

**Supplementary Table 2.** Participant characteristics according to the quartiles of fatty fish

|  | **Fatty fish intake** | | | |
| --- | --- | --- | --- | --- |
|  | **Q1** | **Q2** | **Q3** | **Q4** |
| **Participants**, *n* | 387 | 386 | 386 | 386 |
| **Fatty fish**, g/1000 kcal (median [min–max]) | 3 (0–4.2) | 6 (4.3–7.2) | 9 (7.3–10.9) | 17 (11.0–57.4) |
| **Age**, years | 63.6 (3.6) | 63.5 (3.5) | 63.3 (3.4) | 63.7 (3.5) |
| **Marital status**, married | 89.4 | 92.2 | 92.4 | 88.2 |
| **Education**, <10 years | 4.7 | 4.9 | 3.1 | 7.3 |
| **Employment status**, employed | 70.3 | 72.2 | 72.2 | 71.0 |
| **Household income**, <3 million Japanese yen/year | 30.9 | 28.8 | 25.8 | 35.5 |
| **Current use of cholesterol/triglyceride-lowering medication** | 23.9 | 24.6 | 20.7 | 24.6 |
| **Comorbidities** |  |  |  |  |
| **Diabetes**^1^ | 25.1 | 22.3 | 24.9 | 20.5 |
| **Hypertension**^2^ | 47.8 | 47.2 | 45.3 | 52.6 |
| **Dyslipidemia**^3^ | 60.7 | 60.6 | 57.0 | 57.8 |
| **Cancer** | 6.7 | 4.7 | 7.8 | 7.3 |
| **Chronic kidney disease** | 1.0 | 1.6 | 0.8 | 1.8 |
| **Cardiovascular disease** | 12.4 | 12.7 | 13.0 | 9.3 |
| **BMI**, kg/m^2^ | 24.1 (3.0) | 24.2 (2.9) | 24.2 (3.3) | 24.3 (3.3) |
| **Smoking status**, current | 23.3 | 21.2 | 22.5 | 21.5 |
| **Sleep duration**, <7 hours/day | 37.7 | 42.1 | 35.4 | 34.2 |
| **Physical activity on leisure**, ≥10 METs･h/week | 38.4 | 33.1 | 32.6 | 36.0 |
| **Dietary intake**, per day |  |  |  |  |
| **Total energy**, kcal | 1921 (540) | 1974 (581) | 1806 (344) | 2127 (655) |
| **Protein**, % energy | 13.5 (2.7) | 14.0 (2.5) | 15.1 (2.6) | 16.6 (2.9) |
| **Cholesterol**, mg/1000 kcal | 175.2 (77.8) | 185.6 (68.0) | 199.9 (72.4) | 220.4 (69.7) |
| **Alcohol**, g (median [IQR]) | 9.8 (0.0-31.5) | 14.3 (2.3-31.6) | 9.7 (0.6-28.9) | 14.3 (1.2-31.1) |
| ***n*-3 PUFAs**, g/1000 kcal | 1.1 (0.3) | 1.3 (0.3) | 1.4 (0.3) | 1.8 (0.4) |
| **Vitamin D**, μg/1000 kcal | 4.7 (3.3) | 5.9 (2.8) | 7.6 (3.6) | 10.8 (4.8) |
| **Zinc**, mg/1000 kcal | 4.2 (0.7) | 4.2 (0.6) | 4.4 (0.6) | 4.5 (0.7) |
| **Magnesium**, mg/1000 kcal | 129.7 (28.4) | 133.3 (25.3) | 137.7 (27.2) | 146.1 (29.0) |

Data are presented as percentages or mean (standard deviation) except where noted.

^1^Defined as fasting plasma glucose ≥126 mg/dL, HbA1c ≥6.5%, and/or self-reported medical history or medication use for diabetes.
^2^Defined as systolic blood pressure ≥140 mmHg, diastolic blood pressure ≥ 90 mmHg, and/or self-reported medical history or medication use for hypertension.
^3^Defined as low-density lipoprotein-cholesterol ≥140 mg/dL, triglyceride ≥150 mg/dL, high-density lipoprotein-cholesterol <40 mg/dL, and/or self-reported medical history or medication use for dyslipidemia.
BMI, body mass index; IQR, inter quartile range; MET, metabolic equivalent; *n*-3 PUFAs, omega-3 polyunsaturated fatty acids; Q, quartile; SD, standard deviation

**Supplementary Table 3.** Adjusted means of serum testosterone levels and their 95% confidence interval by quartiles of fish intake among older men, after excluding those with total fish intake of 100 g/1000 kcal or more

|  | **Q1 (*n*=382)** | **Q2 (*n*=381)** | **Q3 (*n*=381)** | **Q4 (*n*=381)** | ***P* for trend**^1^ |
| --- | --- | --- | --- | --- | --- |
| **Total fish**, median (min–max) g/1000 kcal | 14 (0–19.6) | 24 (19.7–29.2) | 36 (29.2–44.1) | 57 (44.2–98.6) |  |
| **Serum testosterone levels** (ng/mL)^2^, adjusted mean (95% CI) | | | | | |
| Model 1^3^ | 5.58 (5.38–5.79) | 5.79 (5.59–5.99) | 5.57 (5.37–5.77) | **5.98 (5.78–6.19)** | **0.04** |
| Model 2^4^ | 5.63 (5.43–5.83) | 5.71 (5.51–5.90) | 5.61 (5.42–5.81) | **5.98 (5.77–6.19)** | 0.07 |
| **Lean fish**, median (min–max) g/1000 kcal | 3 (0–4.2) | 6 (4.2–7.3) | 9 (7.3–11.7) | 18 (11.7–75.5) |  |
| **Serum testosterone levels** (ng/mL)^2^, adjusted mean (95% CI) | | | | | |
| Model 1^3^ | 5.58 (5.38–5.78) | 5.60 (5.40–5.80) | 5.76 (5.56–5.96) | **5.98 (5.77–6.19)** | **<0.01** |
| Model 2^4,5^ | 5.63 (5.43–5.82) | 5.60 (5.41–5.80) | 5.71 (5.51–5.90) | **5.98 (5.78–6.19)** | **0.02** |
| **Fatty fish**, median (min–max) g/1000 kcal | 3 (0–4.2) | 6 (4.2–7.1) | 9 (7.1–10.7) | 17 (10.7–47.1) |  |
| **Serum testosterone levels** (ng/mL)^2^, adjusted mean (95% CI) | | | | | |
| Model 1^3^ | 5.71 (5.50–5.91) | 5.70 (5.50–5.90) | 5.70 (5.50–5.90) | 5.81 (5.60–6.02) | 0.64 |
| Model 2^4,5^ | 5.77 (5.58–5.97) | 5.72 (5.53–5.92) | 5.65 (5.46–5.85) | 5.77 (5.56–5.97) | 0.83 |

The number of participants in each quartile is the same for total, lean, and fatty fish intake. The quartile of fish intake that is significantly associated with serum testosterone levels, with the first quartile as a reference, is shown in bold (*P* value <0.05).

^1^*P* value for trend is calculated by treating the quartiles of fish intake as a continuous term in linear regression model.

^2^Estimated adjusted means and its 95% confidence interval of serum total testosterone level (ng/mL).

^3^Adjusted for age (years, continuous).

^4^Adjusted for age (years, continuous), marital status (married or others), education (<10, 10–12, or ≥13 years), employment status (employed or unemployed), household income (<3, 3–5,9, or ≥6 million Japanese yen/year), body mass index (kg/m^2^, continuous), smoking status (never, former, or current) , use of cholesterol- or triglyceride-lowering drugs (yes or no), alcohol consumption (nondrinkers; drinkers consuming <23, 23–45.9, or ≥46 g ethanol/day), sleep duration (<7, 7–7.9, or ≥8 hours/day), leisure-time physical activity (METs･hours/week, quartile), energy intake (kcal/day, continuous), cholesterol intake (mg/1000 kcal, continuous), zinc intake (mg/1000 kcal, continuous), and magnesium intake (mg/1000 kcal, continuous).
^5^Further adjusted for the combined intake of salted fish and dried fish, small fish with bones, and canned tuna (which were classified as neither lean nor fatty fish), as well as for lean or fatty fish for mutual adjustment.
CI, confidence interval; Q, quartile

**Supplementary Table 4.** Odds ratio for the prevalence of low serum testosterone levels according to quartiles of fish intake

|  | **Q1 (*n*=387)** | **Q2 (*n*=386)** | **Q3 (*n*=386)** | **Q4 (*n*=386)** | ***P* for trend**^1^ |
| --- | --- | --- | --- | --- | --- |
| **Total fish**, median (min–max) g/1000 kcal | 14 (0–19.7) | 24 (19.8–29.4) | 36 (29.5–44.6) | 58 (44.9–162.9) |  |
| Cases, *n* | 26 | 15 | 24 | 16 |  |
| Model 1: OR (95% CI)^2^ | 1 (ref) | 0.56 (0.29–1.08) | 0.92 (0.52–1.63) | 0.60 (0.32–1.14) | 0.29 |
| Model 2: OR (95% CI)^3^ | 1 (ref) | 0.57 (0.29–1.13) | 0.80 (0.43–1.50) | 0.54 (0.25–1.16) | 0.21 |
| **Lean fish**, median (min–max) g/1000 kcal | 3 (0–4.26) | 6 (4.28–7.29) | 9 (7.31–12.1) | 19 (12.2–75.5) |  |
| Cases, *n* | 22 | 26 | 17 | 16 |  |
| Model 1: OR (95% CI)^2^ | 1 (ref) | 1.20 (0.67–2.16) | 0.76 (0.40–1.46) | 0.72 (0.37–1.39) | 0.17 |
| Model 2: OR (95% CI)^3,4^ | 1 (ref) | 1.28 (0.69–2.39) | 0.88 (0.43–1.78) | 0.70 (0.33–1.47) | 0.24 |
| **Fatty fish**, median (min–max) g/1000 kcal | 3 (0–4.2) | 6 (4.3–7.2) | 9 (7.3–10.9) | 17 (11.0–57.4) |  |
| Cases, *n* | 18 | 22 | 22 | 19 |  |
| Model 1: OR (95% CI)^2^ | 1 (ref) | 1.24 (0.65–2.34) | 1.24 (0.65–2.34) | 1.06 (0.55–2.06) | 0.87 |
| Model 2: OR (95% CI)^3,4^ | 1 (ref) | 1.35 (0.69–2.65) | 1.47 (0.73–2.96) | 1.26 (0.59–2.67) | 0.50 |

^1^Calculated by treating the quartiles of fish intake as a continuous term in multiple logistic regression analysis.

^2^Adjusted for age (years, continuous).

^3^Adjusted for age (years, continuous), marital status (married or others), education (<10, 10–12, or ≥13 years), employment status (employed or unemployed), household income (<3, 3–5,9, or ≥6 million Japanese yen/year), body mass index (kg/m^2^, continuous), smoking status (never, former, or current) , use of cholesterol- or triglyceride-lowering drugs (yes or no), alcohol consumption (nondrinkers; drinkers consuming <23, 23–45.9, or ≥46 g ethanol/day), sleep duration (<7, 7–7.9, or ≥8 hours/day), leisure-time physical activity (METs･hours/week, quartile), energy intake (kcal/day, continuous), cholesterol intake (mg/1000 kcal, continuous), zinc intake (mg/1000 kcal, continuous), and magnesium intake (mg/1000 kcal, continuous).
^4^Further adjusted for the combined intake of salted fish and dried fish, small fish with bones, and canned tuna (which were classified as neither lean nor fatty fish), as well as for lean or fatty fish for mutual adjustment.

CI, confidence interval; OR, odds ratio; Q, quartile
